# The epidemiology of injuries related to falling trees and tree branches

**DOI:** 10.1111/ans.17481

**Published:** 2022-01-24

**Authors:** Teagan L. Way, Zsolt J. Balogh

**Affiliations:** ^1^ Department of Traumatology John Hunter Hospital Newcastle New South Wales Australia; ^2^ Discipline of Surgery, School of Medicine and Public Health University of Newcastle Newcastle New South Wales Australia

**Keywords:** climate change, polytrauma, revegetation, trauma, tree related injury

## Abstract

**Background:**

Trees are an essential component of our environment. However, falling trees and/or branches have the potential to cause serious morbidity and mortality. The aim of this study is to describe the nature and severity of tree related injuries based on admissions to a level 1 trauma centre.

**Methods:**

A retrospective review of all trauma admissions related to accidental tree failures was undertaken from January 2013 to June 2021. Patients were identified from the trauma registry using ICD‐10 code ‘W20’. We included basic demographics, hospital admission details and inpatient mortality. Weather information was obtained through the Bureau of Meteorology and remoteness was classified using the Accessibility/Remoteness Index of Australia. Results are presented as mean, standard deviation, median and percentages.

**Results:**

Out of 13 884 admissions, 37 (0.26%) were attributed to trees. 21 (0.15%) of those were injuries due to accidental tree failures and were included in the analysis. 38% were considered to be severely injured based on an injury severity score of greater than 15. 23.8% were polytrauma patients. The chest was the most commonly injured body region (47.6%). Two patients required intensive care admission with ventilator support. The majority of injuries occurred in outer regional areas and 52% of patients were injured when wind speed exceeded 20 km/h.

**Conclusion:**

We demonstrated that the likelihood of being injured by falling trees is very low. This information should be taken into account when planning future developments or considering the removal of existing trees in the interest of public safety.

## Graphical abstract

Large canopy trees are essential part of our environment for healthy and habitable Earth. The epidemiology of tree‐related severe injuries are unknown and often create disproportional media attention. Based on our large population based retrospective study we can conclude that major tree related injuries are negligible in frequency and severity compared to road traffic collisions and comparable in frequency to shark attacks.

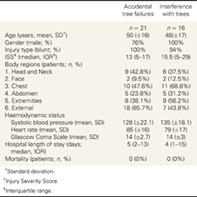

## Introduction

Trees are an essential component of our environment. Every living creature is affected in some way by the world's tree population. The primary function of trees is to provide oxygen and limit carbon in the atmosphere through photosynthesis.^1^ Additional benefits are the reduction in air pollution, providing food and shelter for wildlife, improving soil quality and their ability to lower surface and air temperatures by providing shade and through transpiration cooling. The cooling properties of trees became evident in the 2021 North American heatwave. In areas with denser tree cover, temperatures were said to have been reduced by up to 17°C. Areas such as Vancouver, with little tree cover, saw more heat‐related hospitalisations and mortalities.^2^ However, falling trees or tree branches have the potential to cause serious morbidity and mortality, which generates frequent justification for the removal of trees especially in urban areas associated with new developments. The aim of this study is to describe the epidemiology and the magnitude of injuries related to accidental tree failures, based on the number of trauma admissions to a Level‐1 trauma centre.

## Methods

We conducted a retrospective review of all trauma patients presenting to John Hunter Hospital during an 8.5 year period between January 2013 and July 2021. The John Hunter Hospital has a catchment area of approximately 143 000 km^2^ with a mixed rural and metropolitan population of approximately 1.1 million people.^3^, ^4^

Patients were identified using the ‘W20’ ICD‐10 mechanism code, with a chart review undertaken to identify the patients who were injured due to accidental tree failures. Once patients were identified, data was collected on basic demographics, injury details and location of injury, haemodynamic status on arrival to the emergency department, hospital length of stay (LOS), intensive care length of stay (ICU LOS), ventilator requirements and inpatient mortality. Injury severity score was calculated using the AIS98 coding system. Tree‐related injuries that were sustained during interference with trees, by either professional arborists or individuals without qualification, are described briefly for overall context and non‐statistical comparison purposes, however were excluded from in‐depth analysis.

Weather information was obtained through historical data provided by the Bureau of Meteorology and wind speeds were further categorized using the Beaufort scale.

Remoteness was classified using the Accessibility/Remoteness Index of Australia. Injury post codes were assigned a remoteness index based on accessibility to services.

This project was granted a waiver of consent by the Hunter New England Human Research Ethics Committee (Authorisation Number: AU202107‐29).

## Results

### Sample demographics

Of the 13 884 total trauma admissions to John Hunter Hospital over the study period, there were 37 patients admitted to hospital with tree‐related injuries. Sixteen (0.11%) patients were injured during activities interfering with trees, for example felling and pruning. Those injured during interference with trees had median ISS of 15.5, 94% were blunt by mechanism, all were male, 37.5% required ICU admission and none were fatally injured. Twenty‐one (0.15%) were attributable to accidental tree failures. All mechanisms were blunt type and patients were primarily male. The mean age was 50 (±16) years. Further demographic details for both groups are described in Table 1.

**Table 1:** Demographics and injury characteristics

	Accidental tree failures	Interference with trees
	*n* = 21	*n* = 16
Age (years; mean, SD^†^)	50 (±16)	48(±17)
Gender (male; %)	76%	100%
Injury type (blunt; %)	100%	94%
ISS^‡^ (median, IQR^§^)	13 (5–17)	15.5 (5–29)
Body regions (patients; *n*, %)		
1. Head and Neck	9 (42.8%)	6 (37.5%)
2. Face	2 (9.5%)	2 (12.5%)
3. Chest	10 (47.6%)	11 (68.8%)
4. Abdomen	5 (23.8%)	5 (31.2%)
5. Extremities	8 (38.1%)	9 (56.2%)
6. External	18 (85.7%)	7 (43.8%)
Haemodynamic status		
Systolic blood pressure (mean, SD)	128 (±22.1)	135 (±18.1)
Heart rate (mean, SD)	85 (±16)	79 (±17)
Glascow Coma Scale (mean, SD)	14 (±2.7)	14 (±3)
Hospital length of stay (days; median, IQR)	5 (2–13)	4 (1–15)
Mortality (patients; *n*, %)	0 (0%)	0 (0%)

^†^ Standard deviation.

^‡^ Injury Severity Score.

^§^ Interquartile range.

### Injury characteristics

Of the 21 patients included, eight (38%) had an injury severity score (ISS) of greater than 15 and classified as major trauma. Five out of the 21 patients (23.8%) were polytrauma patients, which is defined as having an Abbreviated Injury Scale (AIS) severity score of equal to or greater than 3 in two or more body regions. After excluding minor soft tissue injuries, the most common body region injured among this group was the chest (47.6%), followed by the head and neck (42.8%). The most frequent injuries are spinal column fractures, followed by multiple rib fractures, pneumothorax and pelvic fractures. Further injury characteristics are described in Table 1.

### Admission details

Of the 21 patients, two were admitted to ICU for 1 and 3 days with both requiring ventilator support. The mean hospital length of stay was 8.9 (±11.6) days. There were no patients who died whilst hospitalized following their injury. Further details are described in Table 1.

### Location of accidents

The majority of injuries occurred in outer regional areas (Table 2). 38% of incidents occurred at home, 33% occurred on farms, 14% occurred in bushland and the remaining 15% occurred between the beach, construction sites and on a footpath.

**Table 2:** Injury circumstances

	*n* = 21
Injury location (patients; *n*, %)	
Major city	5 (23.8%)
Inner regional	7 (33.3%)
Outer regional	9 (42.9%)
Wind speed (patients; *n*, %)	
<4 Beaufort Scale	10 (47.6%)
4–8 Beaufort Scale	10 (47.6%)
>9 Beaufort Scale	1 (4.8%)

### Weather details

Analysis of data provided by the Bureau of Meteorology showed that 52% of patients were injured when wind speed exceeded 20 km/h (Table 2).

## Discussion

Our results have shown that injuries attributed to accidental tree failures make up a very small proportion of the total trauma admissions to our Level‐1 Trauma Centre. When compared to other national and international studies, our findings are not dissimilar.

A study published in 2017, which included all patients with tree‐related injuries showed that 79.4% of admissions were male with the 25–49 age group making up 36.9% of all admissions.^5^ Our study showed similar results in terms of gender however since 2013 the most common age group affected by accidental tree failures is 50–74 years of age.

In terms of injuries, our median ISS was 13, which is higher than previously reported studies from the United States. A study from New Jersey by Hakakian *et al*. however showed similar patterns in terms of common injuries related to tree trauma. The authors reported that head, spine and chest injuries were the most common injuries.^6^

While our hospital did not report any mortalities related to accidental tree‐failures, a paper published using nationwide data examined all Australian deaths from accidental tree failures. The authors identified 51 deaths in a twelve‐and‐a‐half year period. This resulted in an annual mortality rate of approximately 1 in 5 million, based on the average population of 20 million people. Data obtained using the List of Deaths from Accidental Tree Failures in Australia (1858–2019) as well as internet searches for the remaining 2 years (2020–2021) showed that there were 22 prehospital deaths in our local area over the 163 year period, three of which occurred during our study period, with the most recent occurring in November 2021.^7^

Forty‐three percent of these patients were injured in outer regional areas. The Australian Bureau of Statistics^8^ defines outer regional as ‘areas where geographic distance imposes a moderate restriction upon accessibility to the widest range of goods, services and opportunities for social interaction’. Previous studies have not identified location of injury based on geographic location. Walsh & Ryan^5^ commented on patients' residential address rather than injury location stating that only 3% of the Hunter Region residents included in their study population resided in outer regional New South Wales. In terms of place of injury we showed similar results to Walsh & Ryan with the majority of injuries occurring at the patient's place of residence, as opposed to public areas such as roadways, footpaths and bushland.

Weather data supplied by the Bureau of Meteorology provided insight on wind speeds at the time of injury for all patients included in our study population. More than half of our population was injured when wind speeds were in excess of 20 km/h. This equates to a 4 or more on the Beaufort wind scale. The Beaufort wind scale measures wind speed according to impact on land and at sea. At level 4 on the scale, small tree branches begin to move due to wind. One patient in our population was injured when wind was measured at level 10 on the Beaufort scale. This level is rarely experienced on land and has the potential to cause major structural damage.^9^

Due to the dramatic nature of accidental tree failures, these incidents generate a large amount of media attention which has the potential to create unnecessary community hysteria.^5^ Gum trees in particular have sparked debate regarding their appropriateness in urban areas.^10^ Due to their size and tendency to drop limbs, some local governments have ceased planting eucalypts in the interest of public safety. The results from this study as well as others published within Australia and the United Kingdom however suggest that the risk of being injured by an accidental tree failure is far lower than the benefits provided by trees in urban areas.^5^, ^11^, ^12^

Over the years, climate change contributed to an increase in extreme heat events across the globe. A study published in 2019^13^ showed that daytime air temperature decreased with tree canopy cover exceeding 40%. Trees can shade impervious surfaces to reduce heat absorption through the day and also have the ability to cool the air via ‘transpiration cooling’.^14^ This information could be utilized in discussions relating to future town planning.

When compared to other mechanisms such as road trauma, which accounts for approximately 11 000 serious injuries and 300 deaths in New South Wales per year, we can conclude that injury and death related to accidental tree failures is extremely rare.^15^When compared on a local level, the likelihood of being injured due to an accidental tree failure is similar to that of being bitten by shark which accounted for 0.07% of local trauma admissions throughout our study period.

While our study is limited to a single centre involving a small study population, the John Hunter Hospital is the designated trauma centre for a large geographical area so most patients within this area who are injured by a falling tree branch would be transferred to our hospital rather than remaining at local hospitals for treatment. In light of this, the more severe injuries in our study are more than likely overrepresented. Identifying the type of tree responsible for injuries could also be beneficial and larger studies including this information could be useful to further describe the epidemiology of accidental tree failures on a state or nation‐wide level.

## Conclusion

Our findings suggest that the likelihood of being injured or killed by accidental tree failures is very low. Within this overall very low risk scenario, people at the outer regional areas when wind speeds exceed 20 km/h are more likely to be injured. Tree‐related injuries generate a great deal of media attention which has the potential to lead to community hysteria regarding new and existing trees. The extremely low incidence of tree‐related injuries in our study demonstrate that the risk of tree‐related injuries is lower than the known benefits provided by trees. This information should be taken into account when planning future developments or considering the removal of existing trees in the interest of public safety.

## Author contributions

**Teagan L. Way:** Data curation; formal analysis; investigation; methodology; validation; visualization; writing – original draft; writing – review and editing. **Zsolt J. Balogh:** Conceptualization; investigation; methodology; project administration; resources; supervision; writing – review and editing.

## Conflict of interest

None declared.
